# A review of strategies to overcome immune resistance in the treatment of advanced prostate cancer

**DOI:** 10.20517/cdr.2023.48

**Published:** 2023-09-25

**Authors:** Kenneth Sooi, Robert Walsh, Nesaretnam Kumarakulasinghe, Alvin Wong, Natalie Ngoi

**Affiliations:** Department of Haematology-Oncology, National University Cancer Institute, Singapore 119228, Singapore.

**Keywords:** Prostate cancer, immunotherapy, immune checkpoint inhibitor, immune resistance, tumour microenvironment

## Abstract

Immunotherapy has become integral in cancer therapeutics over the past two decades and is now part of standard-of-care treatment in multiple cancer types. While various biomarkers and pathway alterations such as dMMR*, CDK12,* and AR-V7 have been identified in advanced prostate cancer to predict immunotherapy responsiveness, the vast majority of prostate cancer remain intrinsically immune-resistant, as evidenced by low response rates to anti-PD(L)1 monotherapy. Since regulatory approval of the vaccine therapy sipuleucel-T in the biomarker-unselected population, there has not been much success with immunotherapy treatment in advanced prostate cancer. Researchers have looked at various strategies to overcome immune resistance, including the identification of more biomarkers and the combination of immunotherapy with existing effective prostate cancer treatments. On the horizon, novel drugs using bispecific T-cell engager (BiTE) and chimeric antigen receptors (CAR) technology are being explored and have shown promising early efficacy in this disease. Here we discuss the features of the tumour microenvironment that predispose to immune resistance and rational strategies to enhance antitumour responsiveness in advanced prostate cancer.

## INTRODUCTION

Prostate Cancer has the second highest cancer incidence worldwide and is the 5th leading cause of cancer death in men^[1]^. The cornerstone treatment of locally-advanced and metastatic prostate cancer centres upon androgen deprivation therapy. Patients who experience disease progression while having castrate levels of testosterone are considered castration-resistant. In the advanced prostate cancer setting, additional treatment modalities include novel hormonal agents (NHAs), chemotherapy, radioligand therapy, poly(ADP)-ribose polymerase (PARP) inhibitors, and immunotherapy. Successive waves of clinical trials in the past decade have brought these treatment modalities forth from the castration-resistant setting into the hormone-sensitive setting, showing improved survival with early introduction of chemotherapy, NHAs, or combinations of these^[2]^. Despite these advances in prostate cancer treatment, the 5-year survival for metastatic prostate cancer patients in 2022 remains low at 32.3%^[3]^.

Immunotherapy, in the form of sipuleucel-T, received FDA approval in 2010 for the treatment of patients with asymptomatic or minimally symptomatic metastatic castration-resistant prostate cancer (mCRPC). In patients with deficient mismatch repair or microsatellite-high (dMMR/MSI-H) tumours, pembrolizumab and dostarlimab are FDA-approved options^[4,5]^. However, the prevalence of dMMR/MSI-H in prostate cancer is dismal at 1%, with *MSH2* being the most frequently implicated (other MMR genes being *MSH6, MLH1, PMS2*)^[6]^. Owing to an immunologically “cold” microenvironment in unselected acinar prostate adenocarcinoma, to date, no other immunotherapeutic agents have shown to be beneficial in the current treatment of advanced prostate cancer. In this review, we look at the current treatment paradigm, the role of immunotherapy, and existing and up-and-coming methods to overcome immune therapy resistance in prostate cancer.

## IMMUNE REGULATION IN THE TUMOUR MICROENVIRONMENT (TME) OF PROSTATE CANCER

**Figure 1:**
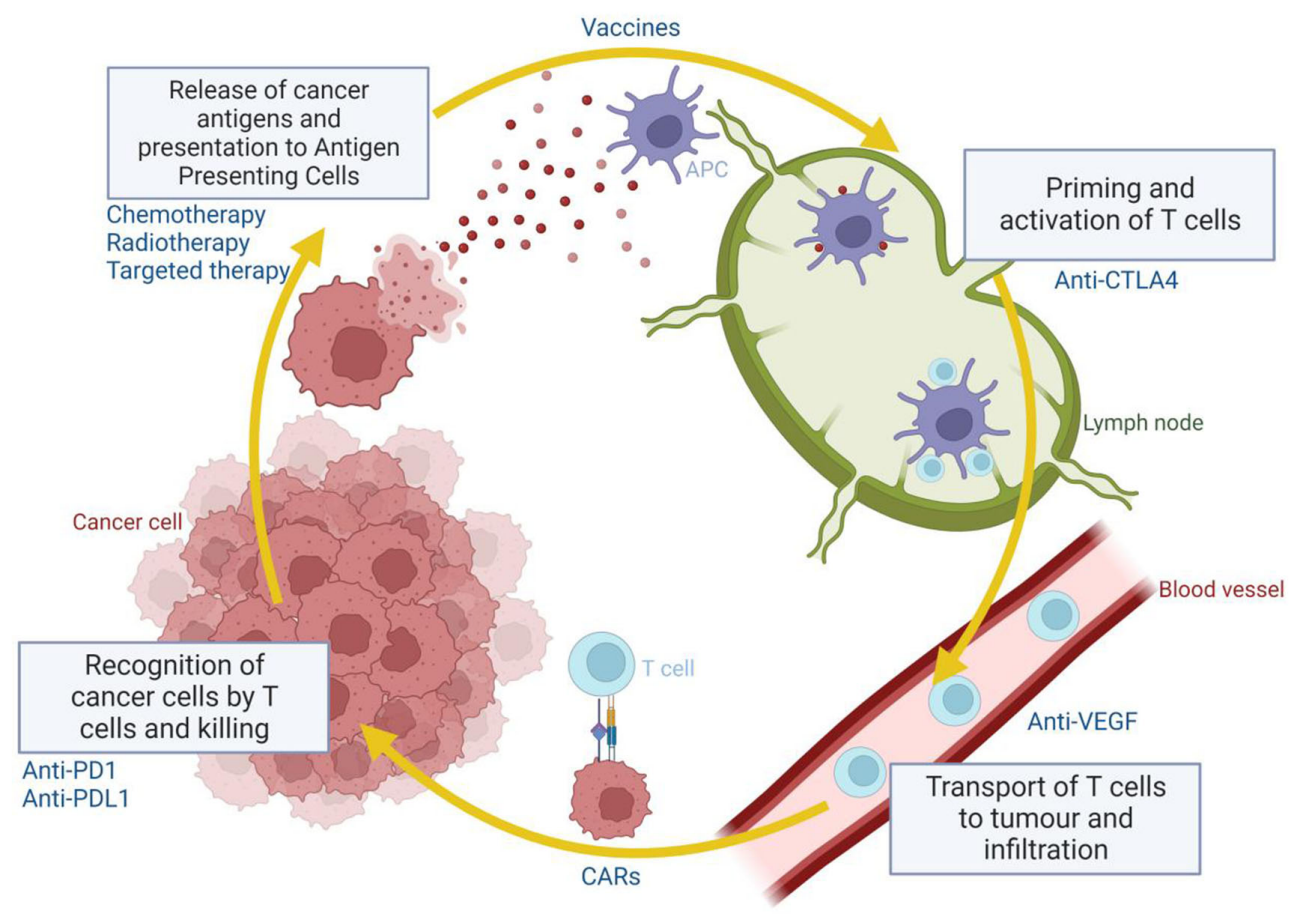
The cancer immunity cycle and where various classes of drugs act on.

Immuno-oncology has changed the treatment paradigm of multiple tumour types, including melanoma, renal cell carcinoma, and lung carcinoma. The cancer-immunity cycle is depicted in Figure 1, explaining how the innate immune system fends off cancer cells and the various points at which therapeutic targets act. Despite successes in these typically immunogenic tumours, prostate cancer has traditionally been considered to have an immunologically “cold” tumour microenvironment (TME) characterized by T cell exclusion, low neoantigen load, and a highly immunosuppressive microenvironment comprising a high proportion of myeloid-derived suppressor cells (MDSCs)^[7,8]^. Factors that suggest a maladaptive immune response against tumour cells include lack of tumour-infiltrating lymphocytes (TILs), presence of M2-polarized tumour-associated macrophages (TAMs) and MDSCs, with evidence that increment in such cell populations within the TME is correlated with tumour progression^[9]^. MDSCs are immune cells that are activated in cancers and display potent immunosuppressive effects leading to prostate cancer resistance to anti-hormonal therapy^[10]^. Furthermore, CRPCs frequently exhibit *PTEN* loss, which is associated with increased MDSC infiltration^[11]^ and may interact with the interferon-1 pathway required for innate immune activation^[12]^. Other immune-suppressive factors within the TME, such as soluble tumour necrosis factor (sTNF), interleukin-1 beta (IL-1β), TGF-β, and IL-10, promote chronic inflammation and increase myeloid cell differentiation into MDSCs^[13,14]^.

**Figure 2:**
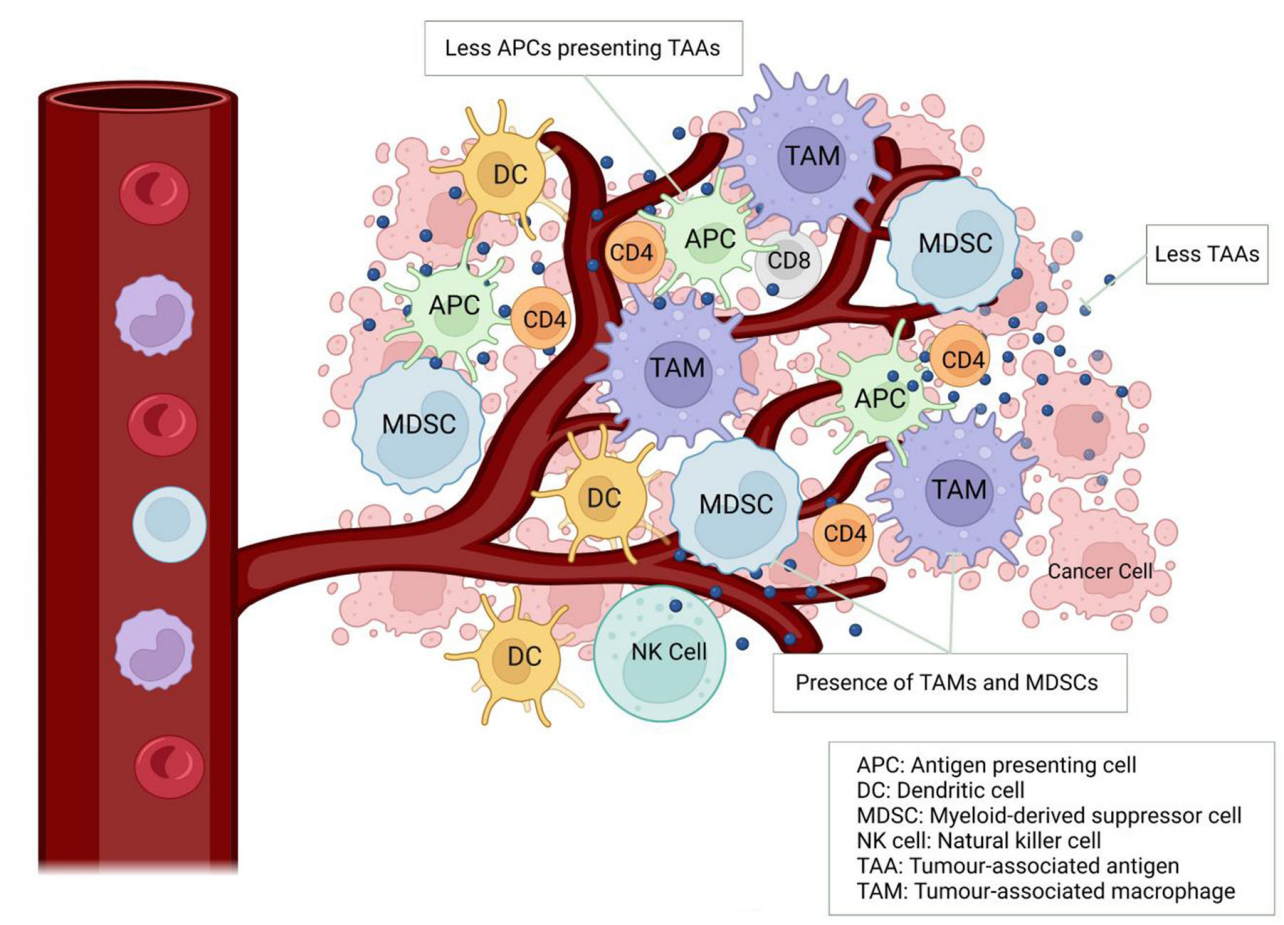
The immunologically “cold” tumour microenvironment in prostate cancer.

Reduced immune stimulatory factors can also contribute to the immunologically cold TME in prostate cancer. CRPC patients have decreased peripheral natural killer (NK) cell pools, and this may be due to increased NK cell group 2 member D (NKG2D) serum receptor levels from the tumour^[15]^. This phenomenon is more pronounced with metastatic disease^[9]^. NK cells are lymphocytes that have roles in innate and adaptive immunity, whereas NKG2D is an activating cell surface receptor expressed on NK cells, NKT cells, and subsets of γδ T cells. Although initially thought to enhance immune responses against cancer, it appears that when NKG2D ligands are expressed chronically, this can instead lead to inhibition of immune cell function^[16]^. Low tumour mutational burden (TMB) in prostate cancer is associated with reduced neoantigen load recognised by the immune system^[17]^. These mechanisms enable immune evasion by cancer cells and directly impact the therapeutic response to anti-PD(L)1/anti-CTLA4 immune checkpoint inhibitors (ICIs)^[18]^. Figure 2 illustrates the interplay amongst the immune cells, cancer cells and vascular supply within the TME.

Potential biomarkers for ICI response include dMMR/MSI-H as mentioned above and tumours with DNA damage repair (DDR) pathway deficiencies. Tumours with DDR pathway deficiencies have increased mutational load as a result of decreased DNA repair capacity, leading to genomic instability^[19]^. Patients with somatic alterations in genes involved in DNA replication or repair have been shown to express higher neoantigen load, higher mutational burdens, higher levels of CD3+ and CD8+ TILs and higher PD-1/PD-L1 levels, all of which correlate with sustained ICI responses^[20-24]^. Despite this, dMMR and *CDK12*-altered prostate cancers have more aggressive biology^[25,26]^. A retrospective study of prostate cancer patients from the Royal Marsden Hospital showed that 8.1% of the patients had dMMR, which was correlated with decreased survival (median OS 4.1 years for dMMR *vs*. 8.5 years for proficient MMR)^[26]^. *CDK12* alterations were found in 6% of advanced prostate cancer in one study^[25]^, and were typically linked to poor prognosis as well as insensitivity to PARP inhibitors^[27]^. However, these tumours have increased neoantigen load and tumoural lymphocyte infiltration, which may increase their response to ICIs^[27]^.

## ICI MONOTHERAPY IN THE UNSELECTED PROSTATE CANCER PATIENT

Cytotoxic T-lymphocyte-associated protein 4 (CTLA-4) is a receptor found on the surface of T lymphocytes. When APCs activate T cells in response to the presence of foreign antigens, there is involvement of costimulatory molecules such as CD28 and B-7, which enhance the immune response. CTLA-4 acts as an immune checkpoint by binding to B-7, counteracting the costimulatory effect of CD28 and overall cause suppression of the immune response^[28,29]^. Cancer cells can downregulate the immune response by exploiting CTLA-4, and this forms the basis of targeting CTLA-4 with monoclonal antibodies such as ipilimumab. Inhibition of CTLA-4 activity causes activation and proliferation of cytotoxic T cells against tumour cells^[30,31]^. To date, two phase 3 trials have looked at the activity of ipilimumab in mCRPC patients. The first study, CA 184-043, recruited 799 mCRPC patients with at least one bone metastasis and have progressed on docetaxel chemotherapy. Patients were randomised to receive either one fraction of bone-directed radiation therapy followed by ipilimumab at 10 mg/kg or placebo. There was no overall survival benefit seen in this study (median OS 11.2 *vs*. 10 months, HR 0.85, 95% CI 0.72-1.00), but a progression-free survival (PFS) benefit (4.0 *vs*. 3.1 months, HR 0.70, 95% CI 0.61-0.82) was seen^[32]^. The second study by Beer *et al.* (2017) randomised 602 mCRPC patients who were chemotherapy-naive and had no visceral metastases to ipilimumab at 10 mg/kg *vs*. placebo. The study showed no overall survival benefit (median OS 28.7 *vs*. 29.7 months; HR 1.11, 95% CI 0.88-1.39), although a PFS benefit (median PFS 5.6 *vs*. 3.8 months; HR 0.67; 95% CI 0.55-0.81) was observed. Exploratory analyses further showed a higher prostate-specific antigen (PSA) response rate with ipilimumab (23%) than with placebo (8%)^[33]^. Taken together, the PFS and PSA response with ipilimumab suggests antitumour activity despite the lack of survival benefit.

PD-1 is a transmembrane glycoprotein found on the surfaces of activated cytotoxic T cells, B cells, dendritic cells, NK cells, and macrophages^[34]^. The binding of PD-1 to its ligands programmed death ligands 1 and 2 (PD-L1 and PD-L2) found on cancer cells delivers inhibitory signals for T-cell activation, suppressing an immune response^[35,36]^. Monoclonal antibodies targeting PD-1/PD-L1, such as nivolumab and pembrolizumab, have shown activity in multiple cancer types, leading to regulatory approval for their use^[37,38]^. Pembrolizumab was studied in the phase 1b KEYNOTE-028 and phase 2 KEYNOTE-199 trials as monotherapy in mCRPC, showing poor responses^[39,40]^. The objective response rate (ORR) was 5% in PD-L1 combined positive score (CPS) ≥ 1 patients in KEYNOTE-199, compared with 3% for patients with a negative PD-L1 expression^[39]^. Three phase 1 dose-escalation trials of nivolumab monotherapy in mCRPC patients likewise showed no objective response^[41-43]^. As mentioned, the paucity of PD-L1 expression in the TME in prostate cancer patients could account for this. Despite the glaringly low response rates for anti-PD(L)1/anti-CTLA4 monotherapies in unselected prostate cancer, the expression of immune checkpoints has been reported to be dynamic, and various agents such as ipilimumab, sipuleucel-T and enzalutamide can increase T cell infiltration into the TME and modulate response to anti-PD(L)1 therapy^[44]^. This sets the stage for combination of various therapies with ICIs to improve immunotherapeutic response in prostate cancer.

## ONGOING STRATEGIES TO OVERCOME IMMUNE RESISTANCE

Several strategies have been examined to modulate antitumour immunity in advanced prostate cancer.

### PARP inhibitors and ICIs

PARP inhibitors are small molecules that prevent the repair of single-strand DNA breaks. Pathogenic DDR gene alterations are found in 23% of mCRPCs^[45]^, with *BRCA2*, *ATM*, *CHEK2*, and *BRCA1* being the most frequently implicated genes^[46]^. The resulting homologous recombination deficiency (HRD) leads to sensitivity to PARP inhibition as a result of synthetic lethality^[47]^. Presently in mCRPC patients, the FDA has approved rucaparib for use in those with germline/somatic *BRCA* mutation and olaparib for those with germline/somatic homologous recombination repair (HRR) gene mutations. This is based on a high ORR of 50.8% seen with rucaparib use in the phase 2 TRITON2 study and improved radiologic PFS with olaparib use over enzalutamide/abiraterone in the phase 3 PROfound study^[48,49]^. The phase 3 TRITON3 study showed similar results^[50]^. Furthermore, efforts made in examining PARP inhibition in unselected patients have been successful as well, with the phase 3 PROpel trial showing improvement in radiologic PFS with combination abiraterone plus olaparib over abiraterone plus placebo as first-line treatment of mCRPC patients, overall suggesting an increasing role in PARP inhibition^[51]^.

Increased micronuclei and cytosolic double-stranded DNA release after PARP inhibitor treatment as a result of PARP-DNA trapping and DNA damage leads to increased neoantigen formation, increased PD-L1 expression, increased intra-tumoural CD8 T cell infiltration and increased interferon production in the TME, forming the basis for ICI-PARP inhibitor combinations, and potentially expanding the benefit of PARP inhibitors beyond tumours harbouring alterations^[52,53]^. A phase 2 open-label clinical trial combining durvalumab with olaparib in men with mCRPC showed a response (radiographic or biochemical) in 9 out of 17 patients. Five of the 9 responders were found to have dysfunctional DDR genes based on genomic analysis and the presence of mutated DDR genes was associated with significantly higher 12-month PFS than those without (83.3% *vs*. 36.4%). Interestingly, patients with fewer peripheral MDSCs were more likely to respond^[54]^. This study showed early evidence of combining PARP inhibitors and ICIs, and other ongoing studies looking at similar combinations are listed in Table 1.

**Table 1:** Trials looking at ICI combinations in treatment of advanced prostate cancer

**Trial number**	**Phase**	**Intervention arm(s)**	**Population**	**Outcome**	**Status**
**ICIs + PARP inhibitor**					
NCT02484404	2	Durvalumab + Olaparib	mCRPC after progression with 1 NHA or Docetaxel	ORR, safety, DOR, PSA response	Completed
NCT04336943	2	Durvalumab + Olaparib	Recurrent prostate cancer with immunogenic signature	PSA response	Active, recruiting
NCT03834519	3	Pembrolizumab + Olaparib NHA (Abiraterone or Enzalutamide)	mCRPC after progression with 1 NHA and chemotherapy	OS, rPFS	Active, not recruiting
NCT02861573	1/2	Pembrolizumab + Olaparib Multiple cohorts	mCRPC	ORR, safety, PSA response	Active, recruiting
NCT05568550	2	Pembrolizumab + Olaparib + RT Pembrolizumab + RT	High-risk localised PC	PSA response	Not yet recruiting
NCT03338790	2	Nivolumab + Rucaparib Nivolumab + Docetaxel Nivolumab + Enzalutamide	mCRPC	ORR, PSA response	Active, not recruiting
NCT04592237	2	Maintenance Cetrelimab + Niraparib Maintenance Niraparib	Aggressive variant mPC given induction Cabazital + Carboplatin + Cetrelimab	PFS	Active, recruiting
**ICIs + vaccines**					
NCT03024216	1	Atezolizumab + Sipuleucel-T	mCRPC	Safety	Completed
NCT01832870	1	Ipilimumab + Sipuleucel-T	CRPC eligible to receive Sipuleucel-T in accordance to FDA-approved labeling	Antigen-specific T cell response, antibody response	Completed
NCT00113984	1	MDX-010 (anti-CTLA-4) + PROSTVAC-V/TRICOM (virus vaccine)	mCRPC after progression with anti-androgens and ≤ 1 chemotherapy	Safety	Completed
NCT02933255	1/2	Nivolumab + PROSTVAC-V/F	mCRPC Neoadjuvant therapy for localised PC planned for surgery	Safety, changes in T-cell infiltration	Active, recruiting
NCT03315871	2	M7824 (anti-PD-L1/TGFβ) + PROSTVAC + CV301 (virus vaccine)	CRPC	PSA response	Active, recruiting
NCT03532217	1	Ipilimumab + Nivolumab + PROSTVAC-V/F + Neoantigen DNA vaccine	mHSPC	DLT, safety, immune response	Completed
NCT03493945	1/2	M7824 (anti-PD-L1/TGFβ) + BN-Brachyury (virus vaccine)+ N-803 (IL-15 superagonist complex) + Epacadostat (IDO1 inhibitor)	CRPC	CBR	Active, recruiting
NCT02325557	1/2	Pembrolizumab + ADXS31-142 (bacteria vaccine)	mCRPC after progression on ≤ 3 systemic therapies	Safety	Unknown
NCT02499835	1/2	pTVG-HP + concurrent Pembrolizumab pTVG-HP + sequential Pembrolizumab	mCRPC	ORR, safety, PSA response, PFS	Active, not recruiting
NCT04090528	2	Pembrolizumab + pTVG-HP (DNA vaccine) + pTVG-AR HP (DNA vaccine) Pembrolizumab + pTVG-HP	mCRPC	PFS	Active, recruiting
NCT04382898	1/2	Cemiplimab + BNT112 BNT112 (RNA vaccine)	mCRPC after progression on 2-3 therapies including NHA and/or chemotherapy	DLT, ORR, Safety	Active, recruiting
**ICIs + tyrosine kinase inhibitors**					
NCT04446117	3	Atezolizumab + Cabozantinib + NHA (Abiraterone or Enzalutamide)	mCRPC after progression on 1 NHA	PFS, OS	Active, recruiting
NCT03170960	1/2	Atezolimab + Carbozantinib	mCRPC after progression on ≤ 1 NHA	DLT, ORR	Active, not recruiting
NCT04477512	1	Nivolumab + Cabozantinib + Abiraterone	mHSPC	DLT	Active, recruiting
NCT04159896	2	Nivolumab + ESK981 (Pan-VEGFR/TIE2 TKI)	mCRPC after progression on 1 NHA and 1 chemotherapy	Safety, PSA response	Unknown
**Combination ICIs**					
NCT04717154	2	Ipilimumab + Nivolumab	mCRPC with immunogenic signature	DCR	Active, recruiting
NCT03570619	2	Ipilimumab + Nivolumab	mCRPC with CDK12 aberration	ORR, PSA response	Active, not recruiting
NCT03061539	2	Ipilimumab + Nivolumab	mCRPC with immunogenic signature after progression on 1 systemic therapy	ORR, PSA response	Active, not recruiting
NCT02985957	2	Ipilimumab + Nivolumab Ipilimumab Cabazitaxel	mCRPC	ORR, rPFS	Active, not recruiting
NCT03333616	2	Ipilimumab + Nivolumab	Non-adenocarcinoma PC	ORR	Active, recruiting
NCT02788773	2	Durvalumab + Tremelimumab	mCRPC with prior exposure to 1 NHA	ORR	Active, not recruiting
**ICIs + androgen receptor antagonist**					
NCT03016312	3	Atezolizumab + Enzalutamide Enzalutamide	mCRPC with prior exposure to 1 NHA and 1 chemotherapy	OS	Completed
NCT02787005	2	Pembrolizumab + Enzalutamide	mCRPC progressing on Enzalutamide	ORR	Completed
NCT04191096	3	Pembrolizumab + Enzalutamide Enzalutamide	mHSPC	rPFS, OS	Active, not recruiting
NCT03834493	3	Pembrolizumab + Enzalutamide Enzalutamide	mCRPC, allows for prior Abiraterone exposure	rPFS, OS	Active, not recruiting
NCT02312557	2	Pembrolizumab + Enzalutamide	mCRPC after progression on Enzalutamide	PSA response	Active, not recruiting
NCT03338790	2	Nivolumab + Rucaparib Nivolumab + Docetaxel Nivolumab + Enzalutamide	mCRPC	ORR, PSA response	Active, not recruiting
NCT01688492	1/2	Ipilimumab + Abiraterone	mCRPC	Safety, PFS	Active, not recruiting
**ICIs + chemotherapy**					
NCT03338790	2	Nivolumab + Docetaxel	mCRPC	ORR, PSA response	Active, not recruiting
NCT04100018	3	Nivolumab + Docetaxel Nivolumab	mCRPC after progression on 1-2 NHAs	rPFS, OS	Active, recruiting
NCT03834506	3	Pembrolizumab + Docetaxel Docetaxel	mCRPC with prior exposure to 1 NHA	rPFS, OS	Active, not recruiting
NCT02861573	1/2	Pembrolizumab + Docetaxel Multiple cohorts	mCRPC	ORR, safety, PSA response	Active, recruiting
NCT03409458	1/2	Avelumab + PT-112 (Platinum + Pyrophosphate ligand)	mCRPC	Safety, PSA response	Active, not recruiting
NCT02601014	2	Nivolumab + Ipilimumab	AR-V7-expressing mCRPC	PSA response	Completed
NCT02788773	2	Durvalumab + Tremelimumab Durvalumab	mCRPC with prior exposure to 1 NHA	ORR	Active, not recruiting
**ICIs + radiopharmaceuticals**					
NCT02814669	1	Atezolizumab + Radium-223	mCRPC after progression on 1 NHA and 1 chemotherapy	ORR, safety	Completed
NCT04109729	1/2	Nivolumab + Radium-223	mCRPC with symptomatic bone metastases	Safety, ctDNA reduction	Active, recruiting
NCT03658447	1/2	Pembrolizumab + 177Lu-PSMA	mCRPC after progression on 1 NHA	Safety, PSA response	Completed

CBR: Clinical benefit rate; CRPC: castration-resistant prostate cancer; DCR: disease control rate; DLT: dose limiting toxicity; DOR: duration of response; ICIs: immune checkpoint inhibitors; mCRPC: metastatic castration-resistant prostate cancer; mPC: metastatic prostate cancer; NHA: novel hormonal agent; ORR: objective response rate; OS: overall survival; PARP: poly(ADP)-ribose polymerase; PC: prostate cancer; PFS: progression-free survival; PSA: prostate-specific antigen; rPFS: radiologic progression-free survival; RT: radionuclide therapy.

As mentioned, *CDK12*-altered prostate cancers typically carry poor prognosis and do not respond well to PARP inhibition, yet they present increased neoantigen load and lymphocytic infiltration, which may increase responsiveness to anti-PD1 therapy^[25,27]^. A retrospective study of 60 men with *CDK12*-altered advanced prostate cancer showed that of the 9 men who received PD-1 inhibitor therapy, 33% had a PSA response and the median PFS was 5.4 months^[27,55]^. Similarly, the ongoing phase 2 IMPACT trial has shown a 21.4% PSA response with ipilimumab-nivolumab combination in these patients^[55]^.

### Vaccines and ICIs

Anti-cancer vaccines can be classified into four groups: cell-based, viral-based, DNA/RNA-based, and peptide-based vaccines^[56,57]^. The goal of vaccine therapy is to stimulate the host’s adaptive immune response against tumour-associated antigens (TAA). Prostate cancer is suitable for vaccine therapy because it has many TAAs such as PSA, prostate-specific membrane antigen (PSMA), prostate acid phosphatase (PAP), prostate stem cell antigen (PSCA), prostate cancer antigen 3 (PCA3), mucin-1, and six-transmembrane epithelial antigens of the prostate (STEAP)^[58]^.

Sipuleucel-T is a therapeutic dendritic cell-based vaccine that has received FDA approval for use in the treatment of patients with asymptomatic or minimally symptomatic mCRPC, based on overall survival (OS) benefit seen from the phase 3 IMPACT trial^[59]^. It is prepared from autologous peripheral blood mononuclear cells obtained by leukapheresis, and pulsed *ex vivo* with PAP2024, a unique fusion protein of granulocyte-macrophage colony-stimulating factor (GM-CSF) and prostatic acid phosphatase (PAP). GM-CSF fosters the maturation of dendritic cells and other APCs to present PAP to the patient’s T cells, resulting in PAP-specific T-cell proliferation targeting the PAP-expressing prostate cancer cells for killing. Both humoural and cellular responses have been reported, with peripheral immune responses to PAP and measures of APC activation correlating with improvements in OS^[60,61]^. Despite success with the use of sipuleucel-T, other vaccines studied have not been as successful. G-VAX is another cell-based GM-CSF-secreting vaccine that utilises irradiated TAAs^[62]^. The TAAs are derived from two cell lines: one hormone-sensitive (LNCaP) and one hormone-resistant (PC3)^[63]^. Despite initially promising results in asymptomatic mCRPC, the phase 3 VITAL 1 and VITAL 2 trials in asymptomatic mCRPC and symptomatic mCRPC patients, respectively, failed to show the OS benefit of G-VAX plus docetaxel against docetaxel alone. Both studies were terminated early based on futility assessments. A viral-based vaccine, PROSTVAC, utilizes recombinant poxviruses that express PSA with immune-enhancing costimulatory molecules to stimulate immune response^[64,65]^. In addition to induced modified human PSA, they contain three costimulatory domains for T cells (B7.1, leukocyte function-associated antigen-3, and intercellular adhesion molecule-1), called TRICOM^[66]^. The phase 3 PROSPECT trial was unable to demonstrate the OS benefit of PROSTVAC against placebo control^[67]^.

Given the increase in T cell infiltration and inflammation within TME with sipuleucel-T^[60,61]^, it is therefore postulated that synergy might be observed with the combined use of vaccines and ICIs. Ipilimumab and PROSTVAC were combined in a phase 1 dose-escalation trial, showing evidence of improved clinical and immunologic outcomes. The median OS was 34.4 months^[68]^, which appears to be numerically larger than PROSTVAC alone in its original study^[67]^. There was a PSA reduction in 54% of patients and a PSA decline of more than 50% was seen in 25% of patients. ADXS31-142 is a live, attenuated, bioengineered listeria-based vaccine targeting PSA. It is being studied as part of the KEYNOTE-046 trial, with current results showing a median OS of 33.7 months for patients treated with combination vaccine and pembrolizumab^[69]^. Other ongoing studies of vaccine therapy with ICIs are listed in Table 1.

### Tyrosine kinase inhibitors and ICIs

Prostate cancers have dysregulated vasculature that promotes an immunosuppressive TME^[7,8]^. These include promoting a shift in TAMs toward M2-like immunosuppressive phenotype, reduced maturation of dendritic cells which results in reduced antigen presentation, and increased PD-L1 expression^[70]^. Vascular endothelial growth factor (VEGF) overexpression has been found to prevent the differentiation of monocytes into dendritic cells^[71]^. Meanwhile, an improvement in the regulation of local vascular in preclinical models was associated with the assimilation of TAMs with M1-like immune-stimulatory phenotype, increased CD4+ and CD8+ T-cell infiltration into the TME, and reduction of MDSCs^[72-75]^. These suggest that targeting angiogenesis in tumours can inhibit tumour-induced dysregulation of local vasculature and promote immunogenicity in the TME, forming the basis of combining antiangiogenesis agents with ICIs. Indeed, it has been shown in renal cell carcinoma that anti-VEGF therapy leads to a reduction in immune inhibitory stimuli such as regulatory T-cells and MDSCs^[76,77]^. Aside from VEGFR targeting, the TAM family of receptor tyrosine kinases comprising TYRO3, AXL and MER has been shown to promote immune suppression as well, making it an attractive target^[78,79]^.

Cabozantinib is a multi-kinase inhibitor targeting MET, VEGFR-1, -2 and -3, AXL, RET, ROS1, TYRO3, MER, KIT, TRKB, FLT-3, and TIE-2^[80]^. Preclinical data suggests that it has an effect on the TME by reprogramming M2 TAMs to “pro-inflammatory” M1 macrophages, in addition to reducing MDSCs and T regulatory cells^[81]^. A dose-expansion cohort in the phase 1b COSMIC-021 trial evaluated the combination of cabozantinib with atezolizumab (anti-PD1) in mCRPC patients who have had disease progression following treatment with novel hormonal agents such as abiraterone or enzalutamide. An ORR of 32% was observed in 132 patients treated with the combination, with a disease control rate (DCR) of more than 80%. This effect was consistent in patients with visceral disease as well^[82]^. Due to promising results from this study, this combination is now being evaluated in a phase 3 clinical trial for mCRPC patients. Other ongoing studies looking at combination anti-VEGF therapy with ICIs are listed in Table 1.

### Combination ICIs

CheckMate-650 is a phase 2 study looking at various dosing combinations of nivolumab with ipilimumab in asymptomatic or minimally symptomatic mCRPC patients who have progressed on novel hormone therapy in two cohorts (chemotherapy-naive and chemotherapy-exposed). In the chemotherapy-naive cohort, nivolumab/ipilimumab achieved an ORR of 25% with a median radiological PFS of 5.5 months and a median OS of 19.0 months. In the chemotherapy-exposed cohort, the ORR was 10%, with a median radiological PFS of 3.8 months and a median OS of 15.2 months^[83]^. Exploratory analyses revealed that PD-L1 ≥ 1%, the presence of DDR or homologous recombination deficiency (if at least one gene in the relevant gene panel had a deleterious mutation/homozygous deletion) were associated with higher ORR^[83]^. In this study, 44 patients had quality-controlled whole-exome sequencing data, giving rise to a median TMB of 74.5 mutations/patient. Tumours harbouring TMB exceeding this median were associated with higher ORR, PSA response rate, radiologic PFS, and median OS^[83]^.

Combination nivolumab and ipilimumab has been examined in AR-V7 expressing mCRPC patients as well. Androgen receptor splice variant 7 (AR-V7) expression is found in approximately 20% of mCRPC patients and is associated with alterations in a greater number of DDR genes, which could increase susceptibility to ICIs^[84]^. The STARVE-PC trial is a phase 2 non-randomised study that evaluated the activity of nivolumab and ipilimumab in 15 AR-V7 expression mCRPC patients, showing an ORR of 25%, PSA response rate of 13% and OS of 8.2 months^[85]^. Responses were more pronounced in six of the patients who were found to have mutations in DDR genes (three in *BRCA2*, two in *ATM,* and one in *ERCC4*)^[86]^. Lastly, an ongoing phase 2 randomised study is looking at mCRPC patients following progression on novel hormonal agents, randomising them to receive durvalumab or combination durvalumab plus ipilimumab. The ORR with combination ICI was 16% *vs*. 0% with durvalumab monotherapy in this study^[87]^. Other ongoing trials evaluating the efficacy of combination ICIs are listed in Table 1.

### Androgen receptor antagonists and ICIs

How prostate cancer treatment impacts the immune response is variable. ADT enhances lymphopoiesis, which can mitigate immune tolerance to prostate cancer antigens^[88]^. On the other hand, androgen receptor antagonists have been shown to inhibit T cell responses^[89]^.

ADT and anti-androgens can both target the AR signalling pathway and have been shown to result in an increase in the number of TILs, and a decrease in the number of regulatory T cells supporting an antitumour response to ADT^[90,91]^. Animal models confirm that while ADT induces pro-inflammatory conditions initially, the subsequent development of castration resistance and immune tolerance to prostate cancer antigens reduces this^[92,93]^. Therefore, the combination of AR-signalling blockade with ICIs, especially during its pro-inflammatory state, may be beneficial in the treatment of advanced prostate cancer.

The phase 2 IMbassador250 trial examined 759 advanced CRPC patients who had progressed on abiraterone and docetaxel, randomising them to receive combination enzalutamide and atezolizumab *vs*. enzalutamide alone. The study was closed prematurely due to futility (combination therapy *vs*. enzalutamide monotherapy, 15.2 *vs*. 16.6 months; HR 1.12, 95% CI 0.91-1.37). However, pre-planned exploratory analyses showed a longer PFS with combination therapy in patients with high PD-L1 IC2/3, CD8 expression^[94]^. The study also performed an unbiased RNA sequencing-based analysis of immune-related gene expression that had previously correlated with mCRPC responses to immunotherapy^[95]^, and found longer PFS with combination therapy in patients harbouring genes related to pre-existing immunity such as TAP-1, CXCL9, interferon signalling^[94]^. The multicohort phase 2 KEYNOTE-199 trial examined combination pembrolizumab with enzalutamide in mCRPC patients whose disease were refractory to enzalutamide. In the cohorts with measurable disease and bone-predominant disease (cohorts 4 and 5), the disease control rate was 51% and ORR was 12%. The duration of response was almost 6 months in 60% of responders^[96]^. This strategy is being evaluated further in an ongoing phase 3 trial [Table 1].

### Systemic chemotherapy and ICIs

Chemotherapy may potentiate antitumour immunity by various mechanisms, including the release of TAAs and enhancing antigen presentation, stimulating the activity of cytotoxic T lymphocytes^[97,98]^. Importantly, chemotherapy may reduce immunosuppressive cell populations such as MDSCs and regulatory T cells, known to maintain prostate cancer immune evasion^[99,100]^. Preclinical studies have suggested that chemotherapy does improve antitumour immune responses, showing that the addition of taxanes can cause a shift in macrophage populations toward the M1-like (immune-activating) phenotype and reduce regulatory T cell and MDSC populations in mouse models^[101,102]^. The multicohort phase 2 trial CheckMate 9KD showed that combination nivolumab and docetaxel in 41 chemotherapy-naive mCRPC patients who have progressed on novel hormonal agents achieved an ORR of 36.8%, radiologic PFS of 8.2 months and PSA response of 46.3%^[103]^. KEYNOTE-365 is an ongoing multicohort phase 1b/2 study examining combination pembrolizumab and docetaxel in mCRPC patients, yielding an ORR of 18%, PSA response of 28%, radiologic PFS of 8.3 months, and OS of 20.4 months^[104]^. Ongoing phase 3 trials (CheckMate7DX and KEYNOTE-921) evaluating the superiority of combination chemotherapy with immunotherapy over chemotherapy alone will shed light in this area [Table 1].

### Radiopharmaceuticals and ICIs

177Lu-PSMA-617 has gained regulatory approval for the treatment of mCRPC patients who have been treated with androgen receptor (AR) pathway inhibition and taxane chemotherapy, based on positive results on a phase 3 trial^[105]^. In murine models, targeted radionuclide therapy (TRT) may increase PD-L1 expression on T cells and the combination of TRT with ICIs leads to increased infiltration of CD8 T cells^[106]^. There is, hence, interest in combining radionuclide therapy with ICIs. Despite low clinical response (ORR 6.8%, PSA response 4.5%, radiologic PFS 3 months) seen on a phase 1b trial combining Atezolizumab and Radium-223 in mCRPC^[107]^, the interim results of another phase 1b/2 PRINCE trial are relatively promising. In this study, 37 mCRPC patients who have progressed on a novel hormonal agent and docetaxel were treated with pembrolizumab and 177Lu-PSMA-617, yielding an ORR of 78%, PSA response of 73%, and 24-week radiologic PFS of 65%^[108]^ [Table 1].

## FUTURE DIRECTIONS AND CONCLUSIONS

Research is ongoing to identify more immunogenic targets and pair them with the multiple TAAs that prostate cancer expresses. Amongst these, cellular-based therapy is an area that deserves special mention. Adoptive cell therapy involves the engineering of patients’ T lymphocytes to target specific viruses or tumours. The use of chimeric antigen receptors (CAR) allows for the creation of artificial T-cell receptors used in adoptive cell therapy^[109]^. A first-in-human phase 1 study of 13 CRPC patients tested PSMA-targeting CAR T cells armoured with a dominant-negative TGF-β receptor. TGF-β is an inhibitory factor found at high levels within the prostate TME. In this study, 4 patients had a ≥ 30% reduction in PSA and 1 patient had a > 98% reduction in PSA. Five patients experienced grade 2 or higher cytokine-release syndrome (CRS)^[110]^. Another CAR T therapy using P-PSMA-101, which targets PSMA, was evaluated in 10 heavily-pre-treated CRPC patients, yielding PSA decline in 7 patients, with 4 patients having > 50% reduction in PSA. CRS was seen in 60% of patients^[111]^. Other CAR T products targeting Epithelial cell adhesion molecule (EpCAM) and Natural Killer Group 2D (NKG2D) have shown activity in prostate cancer patients as well^[112,113]^. Other potential targets of interest with adoptive cell therapy include PSA, PAP, PSCA, and B7-H3^[114]^, and Table 2 shows a list of ongoing clinical trials.

**Table 2:** Trials looking at novel therapies in advanced prostate cancer

**Trial number**	**Phase**	**Intervention arm(s)**	**Population**	**Outcome**	**Status**
**CAR T**					
NCT04227275	1	CART-PSMA-TGFβRDN	mCRPC after progression on 2 NHAs	DLT, safety	Active, not recruiting
NCT03089203	1	CART-PSMA-TGFβRDN	mCRPC after progression on ≥ 1 systemic therapy	Safety	Active, recruiting
NCT04053062	1	LIGHT-PSMA-CART	mCRPC after progression on Abiraterone and chemotherapy	Safety	Suspended
NCT04249947	1	P-PSMA-101 CAR-T	mCRPC	ORR, DLT, safety	Active, not recruiting
NCT03873805	1	Anti-PSCA-CAR-4-1BB/TCRzeta-CD19t-expressing T-lymphocytes	PSCA+ mCRPC	DLT, safety	Active, recruiting
NCT02744287	1/2	BPX-601 (PSCA-specific CAR-T cells)	PSCA+ mCRPC	DLT, safety	Active, recruiting
NCT03013712	1/2	EpCAM-specific CAR T Cells	EpCAM+ mCRPC	Safety	Unknown
**BiTE**					
NCT04104607	1	CC-1 (PSMAxCD3)	mCRPC after progression on ≥ 3 systemic therapies	Safety	Active, recruiting
NCT03792841	1	Acapatamab (PSMAxCD3)	mCRPC after progression on 1 NHA and 1 chemotherapy	DLT, safety	Active, not recruiting
NCT01140373	1/2	HPN424 (PSMAxCD3)	mCRPC after progression on ≥ 2 systemic therapies	ORR, DLT	Active, not recruiting
NCT03972657	1/2	REGN5678 (PSMAxCD28) + Cemiplimab	mCRPC after progression on ≥ 2 systemic therapies	ORR, DLT, safety	Active, recruiting
NCT04221542	1	AMG 509 (STEAP1xCD3)	mCRPC after progression on 1 NHA and 1 chemotherapy	DLT, safety	Active, recruiting
NCT03406858	2	HER2Bi-armed activated T cells (HER2xCD3) + Pembrolizumab	mCRPC	PFS	Active, not recruiting

DLT: dose limiting toxicity; EpCAM: Epithelial cell adhesion molecule; mCRPC: metastatic castration-resistant prostate cancer; NHAs: novel hormonal agents; ORR: objective response rate; PFS: progression-free survival; PSCA: prostate stem cell antigen.

**Figure 3:**
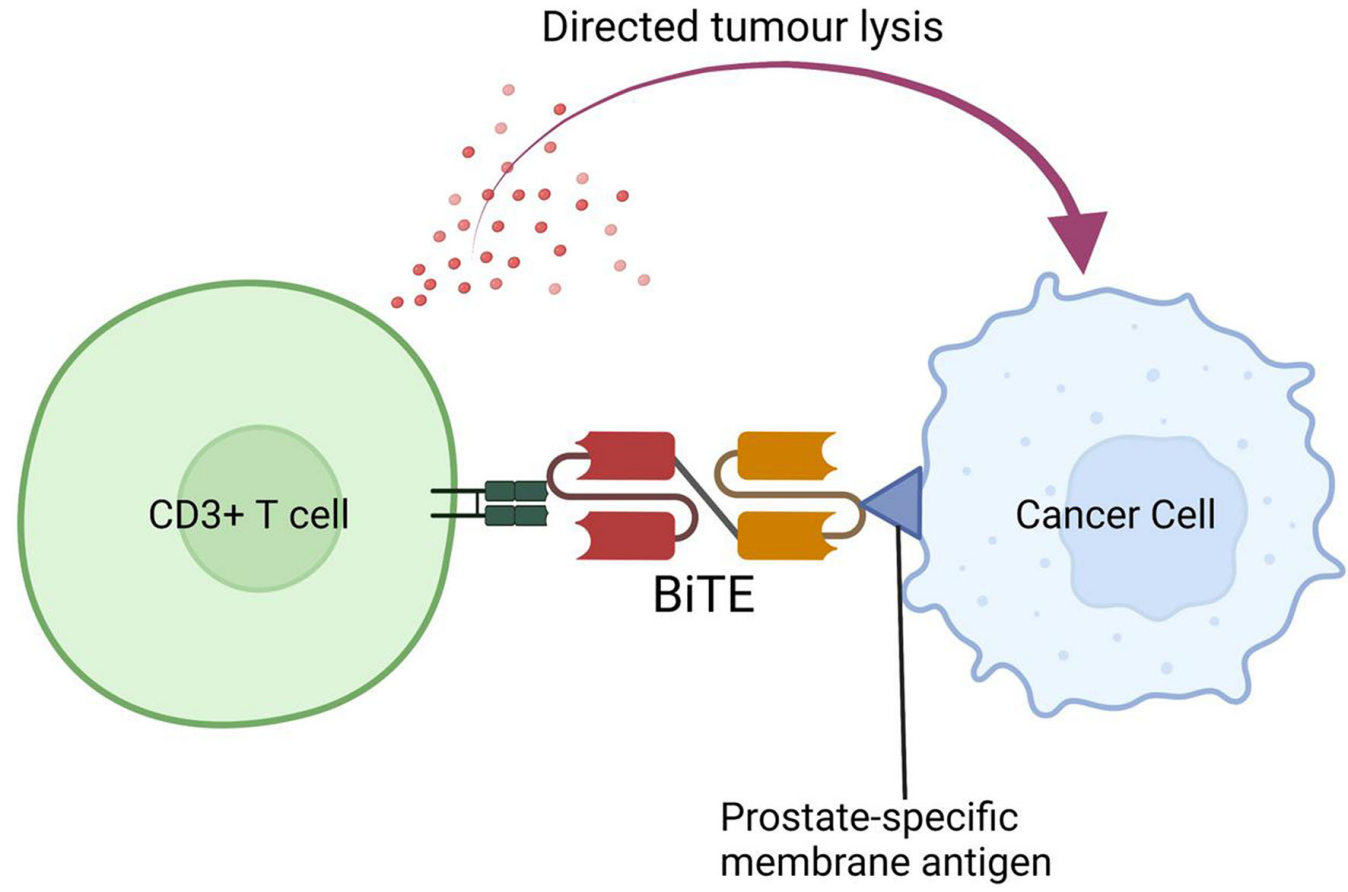
Bispecific T cell engager binding CD3 on T cell with PSMA on prostate cancer cell. BiTE: Bispecific T-cell engager; PSMA: prostate-specific membrane antigen.

Bispecific T cell engager (BiTE) antibodies is another technology that has been developed to target TAAs such as PSMA in prostate cancer cells. Structurally, these are bispecific monoclonal antibodies that can crosslink TAAs with the coreceptors on T cells, generating an antitumour immune response. Pasotuxizumab is a bispecific monoclonal antibody that crosslinks CD3 and PSMA, and its efficacy has been studied in 16 mCRPC patients on a phase 1 trial, showing ≥ 50% decline in PSA in 3 patients, of which two were long-term responders treated for 14.0 and 19.4 months, respectively. 81% of the patients had adverse events of grade ≥ 3^[115]^. The efficacy of AMG 160, a BiTE product that binds CD3 on T cells and PSMA on cancer cells, was evaluated in mCRPC patients on a phase 1 trial. In the preliminary report, 27% of patients had confirmed PSA responses and 84% of patients experienced CRS (10% grade ≥ 3)^[116]^. The study also had a subset of patients who received AMG 160 with pembrolizumab, and such a combination will likely be examined in future studies as well. Other potential BITE targets including STEAP, CEACAM5, DLL3, HER2 are being studied^[117,118]^, and a list of ongoing trials can be seen in Table 2. Figure 3 shows a schematic diagram of BiTE therapy.

On the horizon, relevant and novel targets to modulate antitumour immunity in prostate cancer may include the targeting of relevant immune-metabolic pathways, such as the adenosine receptor^[119-121]^, or cytokine-directed efforts, such as IL-8 involved in the differentiation of TAM to M2 phenotype (promotes immune resistance and tumour metastasis)^[122,123]^, IL-23 which is a cytokine secreted by MDSCs^[124]^ and TGF-β which promotes tumour growth and immunosuppression in the TME^[81]^. Targeting cell signalling pathways such as the phosphoinositide 3-kinase/mammalian target of rapamycin (*PI3K/mTOR*) pathway has also been shown to downregulate immunosuppressive cells such as T regulatory cells and may have a role in improving ICI efficacy in prostate cancer^[125,126]^. For example, in prostate cancer mouse models, intermittent *PI3K* inhibition was able to alleviate *PTEN*-null cancer cell-intrinsic immunosuppressive activity and turn “cold” tumours into T cell-inflamed ones^[127]^. Novel immune checkpoints may be worth exploiting in prostate cancer. Increased expression of V domain Ig suppressor of T Cell activation (VISTA) was found to promote immune resistance following Ipilimumab treatment, which may serve as a new immunotherapeutic target in advanced prostate cancer^[128]^.

There are presently limited biomarkers that can identify prostate cancer patients who may benefit from ICI therapy. It appears that combination strategies to promote immunogenicity within the “cold” TME of prostate cancer can increase the effect of ICIs. We recognise that the majority of the existing efforts are presently in the preclinical or early phase setting and may not be ready for use in the clinics yet. It would nevertheless be interesting to monitor this space for future developments.

## DECLARATIONS

### Copyright

© The Author(s) 2023.
